# A Retrospective Study of Ultrasound-guided Hydrodilatation of Glenohumeral Joint Combined with Corticosteroid Injection in Patients with Frozen Shoulder

**DOI:** 10.2174/0115734056338176241126051407

**Published:** 2024-12-13

**Authors:** Zeng Zeng, Jiang Zhu

**Affiliations:** 1 Department of Ultrasound, Women's Hospital, Zhejiang University School of Medicine, Hangzhou, China; 2 Zhejiang Provincial Key Laboratory of Precision Diagnosis and Therapy for Major Gynecological Diseases, Women's Hospital, Zhejiang University School of Medicine, Hangzhou, China; 3 Cancer Center, Department of Ultrasound Medicine, Zhejiang Provincial People’s Hospital, Affiliated People’s Hospital, Hangzhou Medical College, Hangzhou, China

**Keywords:** Ultrasound-guided, Hydrodilatation, Glenohumeral joint, Corticosteroid injection, Frozen shoulder, Numerical Rating Scale (NRS)

## Abstract

**Objective:**

The purpose of this study was to establish the efficacy of ultrasound (US)-guided hydrodilatation of the glenohumeral joint, in conjunction with corticosteroid injection, in alleviating pain and improving shoulder joint adhesion among patients with primary frozen shoulder (FS).

**Background:**

FS, also known as adhesive capsulitis, is a pathological condition characterized by pain and potential functional impairment. The natural progression of FS involves three distinct stages: freezing, frozen, and thawing. Chronic pain in FS patients can lead to aseptic inflammation, thickening of fibroblasts, and an abundance of type I and III collagen fibers in the vicinity of the glenohumeral joint, ligaments, and tendons. This condition significantly impacts patients' quality of life.

**Methods:**

A total of 200 FS patients were enrolled in this study. All participants underwent US-guided hydrodilatation of the glenohumeral joint, combined with corticosteroid injection, at our department. Pre- and post-treatment (1 year) ultrasound measurements were recorded for the thickness of the axillary recess capsule (ARC), coracohumeral ligament (CHL), and subacromial bursa. Additionally, the numerical rating scale (NRS) and Constant-Murley score (CMS) were assessed to evaluate pain intensity and shoulder function, respectively.

**Results:**

Prior to the commencement of treatment, the measurements indicated a thickness of 4.8±2.3 mm for the ARC, 4.2±1.7 mm for the CHL, and 3.9±1.4 mm for the subacromial bursa. Additionally, the preoperative assessment using the NRS scale for pain yielded a score of 6.4±2.0, while the CMS score for the joint function was 35.8±8.5. Following one year of treatment, a notable decrease was observed in the thickness of ARC, CHL, and subacromial bursa. Furthermore, significant improvements were recorded in both the pain NRS score and the CMS score.

**Conclusion:**

US-guided hydrodilatation of the glenohumeral joint, in combination with corticosteroid injection, may help improve the symptom and function of FS.

## 1 INTRODUCTION

Frozen shoulder (FS) is a common musculoskeletal disease which always causes pain and functional disability [1]. The probability of occurrence before the age of 40 is very low. Frozen shoulder usually occurs around the age of 50 to 60 [2]. It is also called the 50-year shoulder [3]. The prevalence of FS is about 2.5% and it is a self-limiting disease that can be resolved spontaneously and does not require treatment. However, 11% of FS patients suffer from permanent shoulder dysfunction and chronic pain [4]. Chronic pain in FS patients can cause aseptic inflammation and thicken fibro cells and a lot of type I and III collagen fibers around glenohumeral joint, ligaments and tendons [5, 6]. Various histopathologies occur in FS patients, such as inflammation of glenoid, coracohumeral ligament (CHL) hypertrophy, joint capsule fibrosis and so on [7]. FS has three stages: freezing stage, frozen stage and thawing stage. In the freezing stage, some people can recover after conservative treatment [8-10]. Whereas, in the frozen stage, people suffer from pain for decades especially at night time [8]. Although this disease can spontaneously resolve after 2 to 3 years, FS has a very negative impact on people's quality of life. Approximately 40% of patients still have symptoms 4 years after onset and almost 15% of patients have sequelae of long-term disability [11-14]. FS treatment methods include the routine plan of care and intervention comprises surgical and non-surgical treatments [13, 15-17]. To summarize all the methods, the main ones are physical therapy, nonsteroidal anti-inflammatory drugs (NSAIDs), sports rehabilitation, corticosteroid injection, manipulation under anesthesia, surgical ways such as arthroscopic capsular release and manipulation under ultrasound-guided fifth and sixth cervical nerve root block (MUC) [2, 18-21]. In MUC treatment, several complications such as capsule tears, bone bruises, labrum tears in the humeral head, and bone bruises may occur during the block and manipulation procedures [19, 22, 23]. So, the above methods were controversial. There is no consensus which methods is the most effective in FS currently [23, 24].

At present, hydrodilatation represents a significant approach in addressing shoulder contracture [25]. This method involves the administration of normal saline into the glenohumeral joint under the guidance of ultrasound, effectively releasing adhesions within the joint capsule and enhancing joint lubrication. To a notable extent, it alleviates pain symptoms and facilitates rehabilitation processes [26, 27]. In our study, we did ultrasound-guided hydrodilatation of the glenohumeral joint combined with corticosteroid injection treatment. We also did ligamenta coracohumerale release by syringe needle in patients with severe shoulder joint mobility restriction.

Our study conducted a retrospective analysis of 200 patients with frozen shoulder (FS) who underwent ultrasound-guided hydrodilatation of the glenohumeral joint in conjunction with corticosteroid injection in our department. The findings indicate that this method is valuable in ameliorating pain and addressing disorders related to shoulder joint mobility.

## 2 METHOD

### 2.1 Design

This retrospective study was derived from anonymized data in our hospital. It was granted approval by the ethics committee of our hospital (approval no.QT2023004). All of the study protocols adhered to the principles of the Declaration of Helsinki for medical research.

### 2.2 Patients

**Fig. (1):**
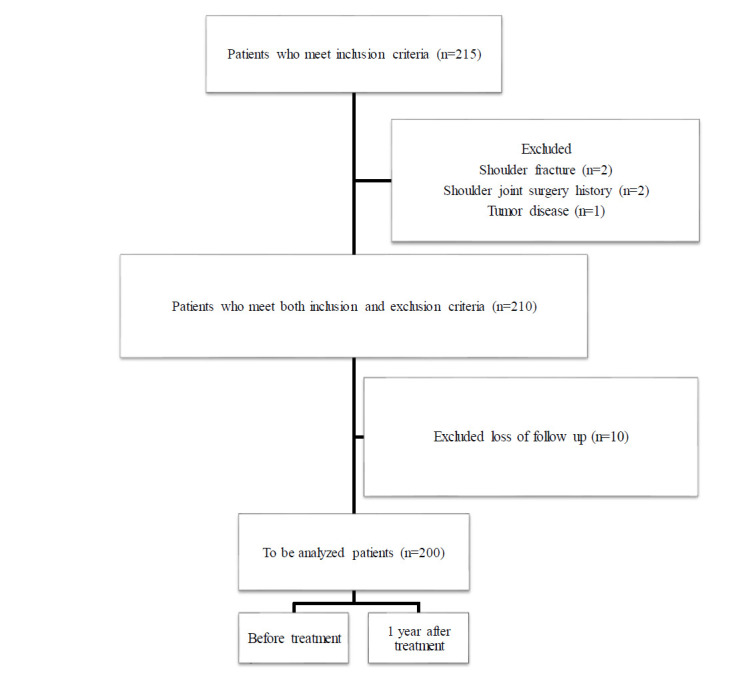
Flowchart diagram.

From June 2022 to June 2023, 215 patients were diagnosed as primary FS. Written informed consent was obtained from all included patients. We collected all the patient's gender, left and right pain, BMI, pain duration, and whether they had diabetes. The inclusion criteria were: (1) Patients had a chronic dull pain in the shoulder. (2) During active and passive movement, patients’ actions such as abduction, flexion, internal rotation, or external rotation of shoulder joints were limited to at least two sets of movement. (3) Ultrasound examination showed a thickened glenohumeral joint capsule (≥2mm). (4) None received any treatment before or after ultrasound-guided treatment. The exclusion criteria were: (1) Shoulder fracture or dislocation. (2) Shoulder joint surgery history. (3) Had diseases such as tumor, rheumatoid arthritis (RA) or tuberculosis. (4) Shoulder area infection (5) Coagulation insufficiency. (6) Secondary FS which was caused by other pathogenic factors. At last, 200 patients were enrolled in this study (Fig. **1**). Patients were followed up for one year after treatment. We also collected the numerical rating scale (NRS) and the Chinese version of the Constant-Murley questionnaire (CMS) both before and after treatment [28, 29]. The NRS score is accurate and concise and was once considered the gold standard of pain assessment by the American Pain Society. The scale is 0-10. 0 means aponia, 1-3 means mild, 4-6 means moderate, 7-10 means severe. CMS was classified into four subscales, including the range of motion (40 points at most), activities of daily living (20 points at most), pain (15 points at most), and strength (25 points at most). A higher total score suggested a higher function of the shoulder (range, 0–100). Scores below 70 should be treated immediately [29].

### 2.3 Musculoskeletal Ultrasound Examination

We used SIEMENS ACUSON Sequoia for ultrasound with high-frequency probe. The patient was in a sitting position. According European Society of Musculoskeletal Radiology-Musculoskeletal Ultrasound Technical Guidelines, we recorded images of long head of the biceps brachii tendon (LHBBT), LHBBT sheath, subscapular tendon, supraspinatus tendon, infraspinatus tendon, teres minor tendon, coracohumeral ligament (CHL), subacromial bursa, rotator cuff space, bursae around the rotator cuff and subaxillary joint capsule. We also paid attention to humeral head bone surface. All ultrasound examinations were conducted by experienced radiologists with 7 years of ultrasound examination experience. For range of motion (ROM), forward flexion, abduction, external rotation and internal rotation of the affected arm were tested both preoperatively and postoperatively. ROM was measured with a goniometer.

### 2.4 Ultrasound-guided Hydrodilatation of Glenohumeral Joint Combined with Corticosteroid Injection Treatment

The ultrasound probe was sanitized. All procedures were performed with the probe covered with surgical gloves. The patient was in lateral decubitus position exposing his/her affected frozen shoulder side on the examination bed. We used complex iodine to disinfect the patient’s skin 3 times and then placed a sterile surgical towel. First, we placed the probe on the lateral side of the shoulder joint. The ultrasound showed both the acromion and the greater tubercle of the humerus. Then, we could clearly see a long axial section of supraspinatus tendon and subacromial bursa. We chose the lateral of the greater tubercle of humerus as the insertion point. We adopted intra-plane injection to do local anesthesia by using 5 mL aliquot of a mixed solution (2.5 mL 2% lidocaine and 2.5 mL 0.9% saline) layer by layer until the subacromial bursa. Lidocaine has low toxicity and a rapid onset. We injected a mixture of 0.4 mL corticosteroid (betamethasone) and then 3 mL saline into the subacromial bursa. In the process of injecting the drug, the subacromial bursa was constantly widened, and the drug was diffused in the front and back of the bursa. Second, the patient laid on the treatment bed with the affected frozen shoulder facing up, and the ultrasound showed the glenohumeral joint capsule. For a clearer display, the patient could also put the affected hand on the shoulder of the unaffected side. We also gave local anesthesia layer by layer until the surface of glenohumeral joint capsule. We injected 0.6 mL corticosteroid (betamethasone) and then 20 mL 2% saline into glenohumeral joint capsule. It is worth noting that the injection flow of drugs in the glenohumeral joint capsule close to the humeral head. It is often easy for inexperienced operators to inject into the infraspinatus. During treatment, the patient’s reaction, such as pain, vasovagal syncope, and so on, should be paid attention to.

### 2.5 Statistical Analysis

We used SPSS 22.0 statistical software (IBM Corp., Armonk, NY, USA) for statistical analysis. The qualitative data were represented by rate and the quantitative data were represented by Mean ± SD. t-test, F-test, chi-square test, or Fisher’s were used to compare differences before and after treatment. *p* value < 0.05 was considered statistically significant.

## 3 RESULTS

Table **1** shows the basic characteristics of FS patients. A total of 200 FS patients (92 males and 108 females) were included in this research. The average age was 56.2±20.4 years old. The number of female patients was a little bit more than male patients. The right shoulder was more easily affected than the left. That may be because most Chinese individuals had the right dominant limb. These patients' pain duration was 9.3±4.8 months. Table **2** shows a comparison of results before and 1 year after treatment. Before treatment, the thickness of ARC was 4.8±2.3 mm, the CHL thickness was 4.2±1.7 mm, the thickness of the subacromial bursa was 3.9±1.4 mm, the preoperative pain NRS was 6.4±2.0 and the joint’s CMS score was 35.8±8.5. One year after treatment, there were significant differences in every outcome value in Table **2** (All *p* < 0.05). The thickness of ARC, CHL, and subacromial bursa all decreased significantly. Both the pain NRS score and the joint’s CMS score improved 1 year after treatment. There were no severe adverse complications, such as infection or bleeding; some patients feel a little bit of injection site swelling.

Fig. (**2**) shows the main procedures of diagnosis and treatment. In FS patients, the glenohumeral joint capsule was significantly thickened. The subacromial bursa was widened after injection under US guidance. Saline mixed with a few air bubbles was injected under US guidance in the posterior recess bursa of the glenohumeral joint to do hydrodilatation. We can see the flaky hyperechoic area due to the reflection of air bubbles. Compared with traditional surgery, this method is very patient-friendly, does not need hospitalization and only has two needle holes.

Fig. (**3**) shows the range of motion (ROM) (forward flexion/abduction/external rotation/internal rotation) at pre-treatment and 1 year after treatment. The degrees of ROM in forward flexion (89 [80-105] at baseline to 155 [150-165] at 1-year follow-up), abduction (125 [110-135] at baseline to 170 [155-180] at 1-year follow-up), external rotation (10 [5-15] at baseline to 50 [40-60] at 1-year follow-up) and internal rotation (35 [20-40] at baseline to 50 [45-60] at 1-year follow-up) showed improvement significantly (All *p* < 0.001).

## 4 DISCUSSION

In our research, the utilization of ultrasound-guided hydrodilatation of the glenohumeral joint in conjunction with corticosteroid injection has proven to be an efficacious treatment modality for patients with frozen shoulder (FS). Our methodology encompasses not only the employment of musculoskeletal ultrasound for the purpose of observing the shoulder joint in FS patients but also the comprehensive utilization of ultrasound guidance during the therapeutic process. Sasanuma *et al*. evaluated positive findings of capsule tears, labrum tears and bone bruises of humeral head of severe idiopathic frozen shoulder patients by magnetic resonance (MR) imaging [23]. MR is more expensive than in the US, and the examination takes a longer time. FS patients have US findings such as thickening of the coracobrachial tendon, coracoacromial ligament, subaxillary joint capsule and scapulohumeral periarthritis [30, 31]. Through ultrasonic guidance, we can clearly see the position of the needle tip in real-time dynamics so as to achieve precise treatment. Patel *et al*. also found ultrasound-guided injections are more accurate at reaching the glenohumeral joint than freehand injections from a cadaveric study as well [32]. The advantage of our study is that under real time ultrasound guidance, we place the needle in a safe zone far enough from any vital structure and avoid injury.

Many studies also indicate that in primary FS, the ROM of the shoulder joint can improve after US-guided shoulder joint injection [33]. Galluccio *et al*. did a study and found that shoulder anterior capsular block, alone or in combination with other blocks can relieve shoulder pain [34]. Xu *et al*. demonstrated that manipulation under anesthesia can also improve function and relieve pain of secondary frozen shoulder (SFS) [35]. SFS means having a definite etiology leading to shoulder stiffness, for example, trauma or surgery [35, 36]. In our study, all FS patients’ pain and limited mobility improved to varying degrees, indicating that this treatment is effective. ROM of forward flexion, abduction, external rotation and internal rotation at pre-treatment and 1 year after treatment showed improvement significantly (All *p* < 0.001).

The reason for FS is unclear. Continuous tension in patients’ posture and increasing inflammatory factors may cause FS [24, 37]. FS has freezing stage, frozen stage and thawing stage [38]. While studies have demonstrated that frozen shoulder (FS) is a self-limiting condition, it is noteworthy that a subset of patients may experience prolonged pain during the frozen stage. This prolonged pain significantly impacts the quality of life of these patients. Researchers have, therefore, identified multiple avenues for addressing chronic pain associated with musculoskeletal disorders. For example, high-dose oral steroids, massage, acupuncture, extracorporeal shockwave therapy, percutaneous electrical nerve stimulation, the release of the shoulder joint cavity under brachial plexus block, the arthroscopic release of the shoulder joint capsule and continuous radiofrequency [6, 39, 40]. However, the effectiveness of these interventions is not satisfactory for patients. Some patients still have pain or refractory pain after these treatment methods. For example, steroid drugs can control pain, but they only have short acting time [39-41].

The etiology and pathological mechanism of primary frozen shoulder (PFS) are complex and numerous. There is no study that clarifies the mechanism of FS. Lho *et al*. studied the fact that elevated levels of inflammatory cytokines in the subacromial bursa may cause inflammation [42]. The normal volume of glenohumeral joint lumen is about 15-20 mL; however, in FS patients the volume is always less than 10 mL [26]. The flexibility of the bursa fold is always caused by the proliferation and fibrosis of axillary recess capsule (ARC) of the glenohumeral joint. The ARC thickness of FS patients in our study is 4.8±2.3 mm, which is thicker than that of normal people. The limited ROM of shoulder is related to the proliferation of anatomical structure fibers and fibrosis of the ligament [5, 43]. Contracture, adhesion and inflammation of the anterior joint capsule, for example, CHL can affect external rotation, extension and abduction of the shoulder [44]. In frozen stage, the thickness of CHL has negative correlation with external rotation and internal rotation ranges of the shoulder [43]. The external rotation is the most obvious limitation of FS [43]. In our research, the CHL thickness is 4.2±1.7 mm, which is thicker than normal people. Xu *et al*. found that FS patients with thickening of the CHL did not receive good improvement of shoulder ROM when using US-guided distension of the shoulder joint cavity alone [6]. In our study, CMS of the shoulder joint, abduction, external rotation, internal rotation and forward flexion can be significantly improved after treatment. Internal rotation is a compound action; severe fibrosis and adhesions of the shoulder joint have a negative impact on recovery. Ultrasound-guided hydrodilatation of glenohumeral joint combined with corticosteroid injection can solve this problem as well. ROM of internal rotation improved from 35 [20-40] at baseline to 50 [45-60] at 1-year follow-up in our study.

The shoulder joint cavity is about 15-20ml, however, in FS patients, the volume is always reduced because of intraarticular synovial contracture. Studies have shown that using sterilized water is effective in shoulder joint capsule distension [45]. We injected 20ml saline into the glenohumeral joint capsule, and therefore, the adhering joint cavity mechanically separated. In some patients with severe shoulder mobility disorder, we also used acupuncture to loosen the coracohumeral ligament to improve the adhesion of the shoulder joint. After shoulder joint expansion, the joint capsule contracture and the ROM can be improved. We suggested that FS patients should do arm climbing training slowly after treatment twice a day to enhance shoulder joint function. Therefore, we have optimized the treatment to better improve the patients with shoulder pain and mobility disorders.

In FS patients, there is always inflammation, which can be detected by US findings of effusion and reduced tendon clearance space. Many studies have demonstrated that steroids can effectively control synovitis [40, 41]. Corticosteroid has anti-inflammatory effect and can inhibit inflammation [46]. This is the reason we inject corticosteroid. It is a medium to long-acting hormonal drug. It is worth noting that we should shake it well before the injection. The compound betamethasone is a lipid-soluble granular suspension and will limit the drug from being applied evenly on the synovial membranes or in the interstitial spaces with adhesions.

Mens *et al*. found that injecting a mixture of lidocaine, triamcinolone and air into shoulder capsulitis with hearing squishing sound showed accuracy to efficacy [47]. We suspect that this is because this squishing sound means that the joint cavity is stretched open. In our research, we not only hear this squishing sound but also observe a flaky hyperechoic area due to the reflection of air bubbles. This hyperechoic area must be seen adjacent to the humerus to ensure a mixture of medicine is injected into the shoulder capsulitis. It is worth noting that for inexperienced operators, it is often easy to inject into the infraspinatus.

There are several limitations in this study. First, the follow-up time is 1 year, long-term follow-up of these FS patients should be performed. Second, there exists a bias in personal pain sensitivity. Some people tolerate pain better. No control group was the third limitation of this study, making it difficult to rule out the effects of the disease’s natural self-resolution and the placebo effect of treatment. All the above need to be further studied.

## CONCLUSION

Ultrasound-guided hydrodilatation of glenohumeral joint combined with corticosteroid injection may relieve the pain of primary FS patients, improve the adhesion of shoulder joint, and show value in clinical.

## Figures and Tables

**Fig. (2):**
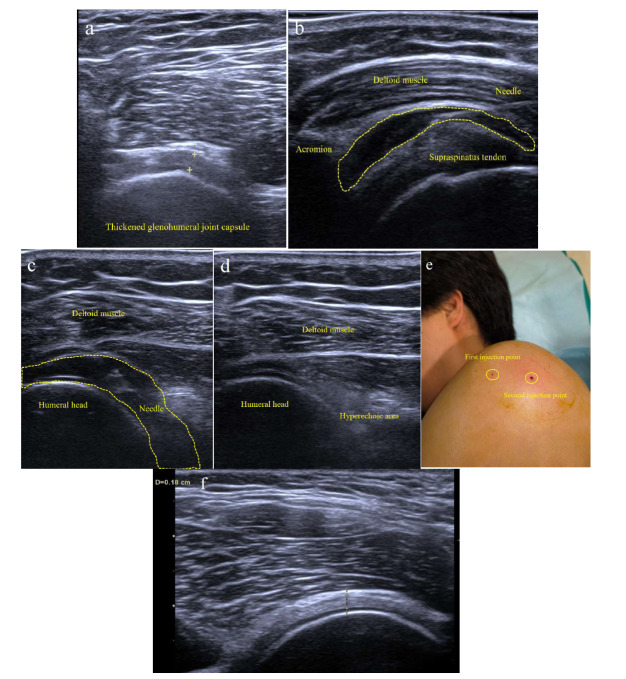
**a**) Ultrasound images for diagnosing FS. The glenohumeral joint capsule was significantly thickened (0.30cm). **b**) Subacromial bursa was widened after injection under US guidance (anechoic area rounded by yellow dotted line). **c**)Saline mixed with air bubbles were injected under US guidance in the posterior recess bursa of the glenohumeral joint to do hydrodilatation. Glenohumeral joint was widened (anechoic area rounded by yellow dotted line). **d**) Flaky hyperechoic area can be observed due to the reflection of air bubbles. **e**) After the treatment, only two tiny needle eyes can be seen. **f**)3 months after treatment, normal glenohumeral joint capsule (0.18cm).

**Fig. (3):**
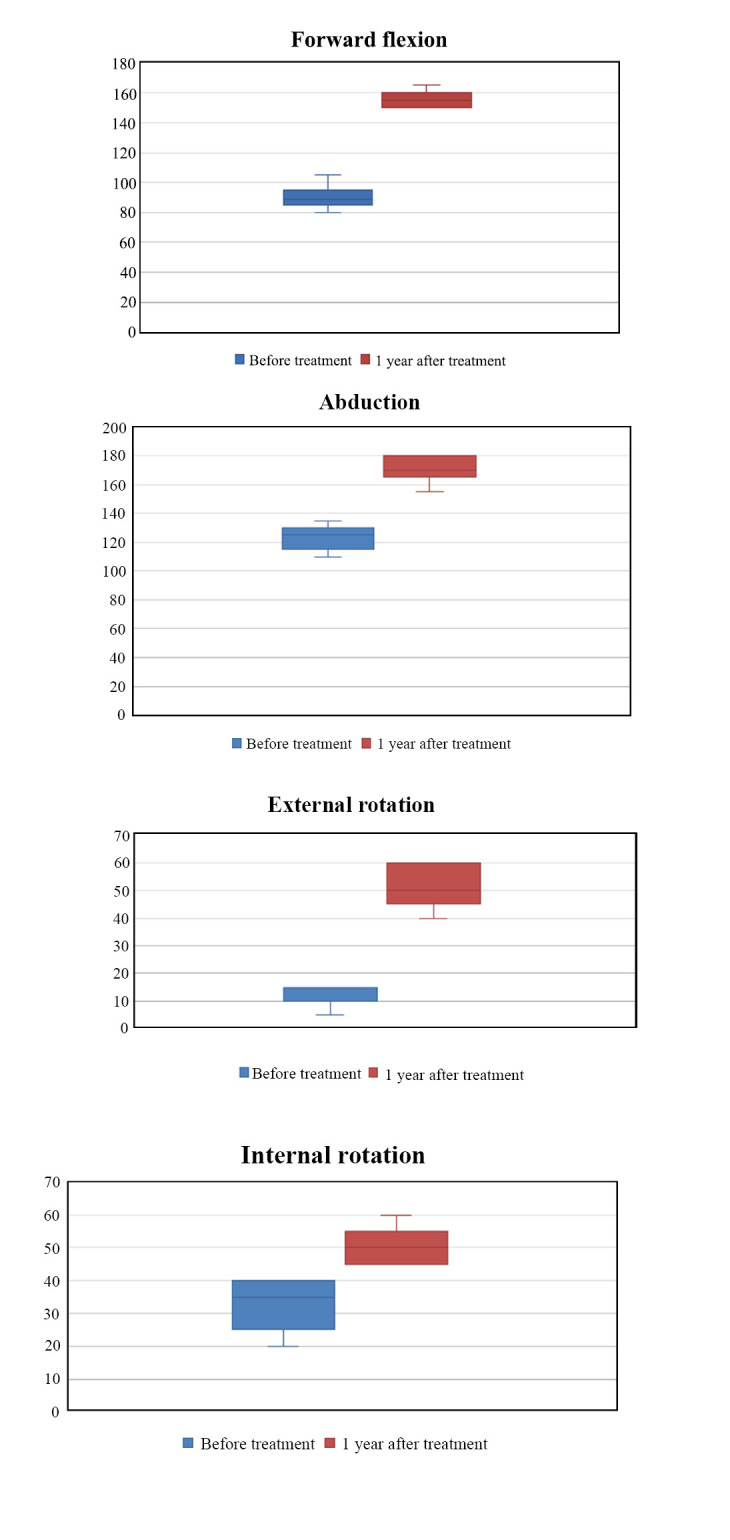
Each outcome value (forward flexion/abduction/external rotation/internal rotation) at pre-treatment and 1 year after treatment.

**Table 1:** FS patient’s demographics at baseline.

Characteristics	-
Age	56.2±20.4
Male/Female	92/108
Pain side (Left/Right)	89/111
Body mass index (kg/m2)	20.4±9.8
Diabetes	45
Duration of pain (months)	9.3±4.8

**Table 2:** Comparison of results before and 1 year after treatment.

-	Before Treatment	1 year after Treatment	*p* value
Thickness of ARC	4.8±2.3	1.8±0.5	<0.001*
Thickness of CHL	4.2±1.7	2.1±1.1	0.013*
Thickness of subacromial bursa	3.9±1.4	1.7±1.0	0.042*
NRS score	6.4±2.0	2.5±2.2	<0.001*
CMS score	35.8±8.5	81.3±10.9	<0.001*

**Abbreviations: **ARC: Axillary recess capsule; CHL: Coracohumeral ligament; NRS: Numerical rating scale score; CMS: Constant-Murley score.

## Data Availability

All data generated or analysed during this study are included in this published article.

## LIST OF ABBREVIATIONS

CMS: Constant-Murley Score
LHBBT: Long Head of the Biceps Brachii Tendon
ROM: Range of Motion
FS: Frozen Shoulder
US: ultrasound
ARC: Axillary Recess Capsule
CHL: Coracohumeral Ligament
NRS: Numerical Rating Scale

## STANDARDS OF REPORTING

STROBE guidelines were followed.
